# Predictors of femoral shortening for pediatric developmental hip dysplasia surgery: an observational study in 435 patients

**DOI:** 10.1186/s13037-018-0176-y

**Published:** 2018-10-19

**Authors:** Nabil Alassaf

**Affiliations:** Department of orthopaedic surgery, King Fahad Medical City, P.O.BOX 59046, Riyadh, 11525 Kingdom of Saudi Arabia

**Keywords:** Hip dislocation, Complications, Child, Osteotomy

## Abstract

**Background:**

Open reduction of the congenitally dislocated hip may not be possible without femoral shortening. The goal of this study is to develop a prognostic prediction model for the need of femoral shortening in children undergoing anterior open reduction for the treatment of developmental dysplasia of the hip (DDH). The secondary objective was to determine if femoral shortening influences the risk of adverse events.

**Methods:**

A cohort from February 1, 2008 thru July 31, 2017 was studied retrospectively at a single centre. Patients between the age of 1 and 8 years, having international hip dysplasia institute (IHDI) grade 3 and 4, undergoing primary anterior open reduction for DDH were included in the study. The outcome of interest was femoral shortening, and the potential predictors were age, sex, side, body mass index and IHDI grade. Logistic regression was employed to identify the independent predictors and was followed by internal validation using bootstrapping. In addition, complications encountered were recorded and analysed.

**Results:**

A total of 548 hips in 435 patients were included. Median follow-up (interquartile range) was 27 (13–48.25) months. Femoral shortening was needed on 119 hips. Factors that increased the probability of femoral shortening in the reduced model were age, male gender, and IHDI grade 4. Adjusting for IHDI and the addition of pelvic osteotomy, the probability of recurrence was lower when femoral shortening is included and higher with increased patient age. There were more deep infections when femoral shortening is added. Femoral shortening did not affect the occurrence of avascular necrosis.

**Conclusion:**

In addition to age and superior displacement of the femoral head, male gender is considered to be an independent predictor for needing femoral shortening. Studying the probability of femoral shortening in DDH surgery may optimize family education, operating room preparation, and operative time utilization. Moreover, there appears to be less risk of recurrence when femoral shortening is performed at the cost of higher probability of deep surgical site infection.

## Background

Femoral shortening is a fundamental component of the surgical treatment of developmental dysplasia of the hip (DDH) and usually considered on an individualized basis. Surgeons use femoral shortening to make open reduction possible when the soft tissues are significantly contracted and also occasionally added to alleviate pressure on the reducible femoral head. Excessive tension across a reduced hip may increase the risk of avascular necrosis, redislocation and probably stiffness [1–3]. With few exceptions, femoral shortening is most widely accomplished through an additional lateral surgical exposure [4]. Ombredane was the first to described subtrochanteric femoral shortening in combination with the open reduction to treat congenital hip dislocation in children [5]. In the past, femoral shortening was considered only for patients who did not respond to prolonged preoperative traction [6]. An alternative to skeletal traction is an external distractor, which Wojciechowski et al. used to lower the femoral head before open reduction and acetabuloplasty [7]. This method, however, entails two-stage surgery and carries the additional risk of pin site infection. Earlier, Grill used this technique to avoid femoral shortening in children who were older than 6 years of age [8]. In an analysis of 39 hips, Schoenecker et al. did not find an advantage for preoperative traction in patients older than 3 years of age, particularly, for the reduction of avascular necrosis rate [1].

Subtrochanteric rather than intertrochanteric osteotomy, is commonly used in femoral shortening. Ashley et al. suggested that the amount of shortening required is the overlap between the femoral shaft and the proximal fragment, which usually measures 2 to 3 cm [9]. They fixed the two pieces using a four-hole plate without changing the rotation. Shih et al., in a study of 20 hips in children between the age of 2 and 11, had no shortening that exceeded 2 cm [10];there were one redislocation and one resubluxation, both believed to be secondary to the overcorrection of femoral head anteversion. Galpin et al. reviewed 33 hips in children over the age of 2 who had open reduction and femoral shortening; there was no symptomatic leg length discrepancy [11]. As we are beginning to understand the pathoanatomy of DDH further, there are fewer varus producing proximal femoral osteotomies performed today as persistent femoral neck varus may result [2]. Rotating osteotomies to improve anteversion, which is occasionally severe in DDH patients, continue to be only cautiously used. Sankar et al. further studied the effect of age and femoral head migration on the overall rate of femoral shortening [12].

When deemed useful, femoral shortening require extra operative time and equipment, and it may also increase the need for the administration of blood products. Being informed about the probability of femoral shortening beforehand will help enlighten the patient’s family as well as the healthcare providers about this surgical step. The goal of this study, therefore, is to develop a prognostic prediction model for the need of femoral shortening in children who are undergoing anterior open reduction for the treatment of DDH. The secondary objective is to examine whether or not femoral shortening alters the risk of adverse events.

## Methods

### Patients

This observational study was approved by an institutional review board, and conducted in compliance with the Declaration of Helsinki. A prognostic model development, retrospective cohort study design. A database in a single tertiary care facility was queried for consecutive patients between the first of February 2008 and the end of July 2017. Eligibility criteria were primary surgery for DDH, ages between 1 and 8 years at the time of operation, undergoing open reduction through an anterior approach, and international hip dysplasia institute (IHDI) grades 3 and 4. Exclusion criteria were non-idiopathic hip dislocation secondary to local or systemic diseases, reoperation, patients less than 1 years or older than 8, as these patients are typically treated with a different surgical protocol. Open reductions through medial approach and IHDI grade two or less were factors that were not analyzed.

### Surgical procedure

The decision to proceed with femoral shortening was based on intraoperative assessment without a cut-off age, namely, either the inability to reduce the hip or the hip being reducible, but under excessive tension. After anterior exposure and capsulotomy of the hip, femoral shortening was performed through a separate incision, using a standard lateral approach to the femur. Adductor longus, rectus, and iliopsoas tenotomies were performed routinely. The amount of shortening varied among the patients. Two transverse subtrochanteric osteotomies are done, and a 4-holes plate is used in addition to immobilization with a hip spica. External rotation of the distal fragment was not done in all patients and instead reserved for hips with marked anteversion compromising stability. A pelvic osteotomy is then added after femoral shortening, followed by capsular repair. If the dislocation is bilateral, the other hip is scheduled on a different day. No preoperative traction was used.

### Outcome and predictors

The addition of femoral shortening during open reduction in primary pediatric DDH surgery was the dichotomous outcome of interest. The medical records and radiographs were examined for demographic parameters and surgical details. The following potential preoperative predictors were extracted and included in the analysis: Age in months, sex, side of the operated hip, Body mass index (BMI), and severity of hip displacement as outlined using the IHDI classification. Data pertaining to complications were collected and compared between hips that had femoral shortening and those who did not. The risk of recurrence and avascular necrosis were analysed while controlling for the following parameters: femoral shortening, age, pelvic osteotomy, and IHDI grade. Recurrence was defined by persistent disruption of the Shenton-Menard line and avascular necrosis was determined based on Salter’s criteria [13].

### Data analysis

The full model included five predictors. A sample that included more than 50 events of femoral shortening was felt to be adequate according to the rough 1:10 rule [14]. Five multiple imputations were performed for missing values at random. For univariable analysis, the normality of distribution and homogeneity of variances for the continuous variables were determined graphically. Mann-Whitney-Wilcoxon test was used in the absence of normality, and an independent Student’s t-test was used for the parametric values. Chi-square test and Fisher’s exact test were used for count data. In the multivariable analysis, the predictors’ selection was done by backwards eliminating binary logistic regression using *p*-value. The stopping rule was the Akaike information criterion (AIC). Fractional polynomials were used to handle continuous variables during model selection. For internal validation, model discrimination was computed using 100 samples, the area under the curve (AUC) was calculated and the receiver operator characteristic (ROC) curve was plotted. The variance inflation factor (VIF) was obtained for each predictor to check for multicollinearity with a value of 10 or more set as high. To correct for optimism, the shrinkage factor was calculated through 200 bootstraps. After shrinkage, the calibration slope was calculated and illustrated using 40 repetitions. A *p*-value of less than 0.05 was considered significant in the univariable comparisons. R software was used for the statistical calculations, Version 3.4.3 (R Foundation for Statistical Computing, Vienna, Austria).

## Results

In total, 548 hips in 435 patients were eligible for inclusion. Forty-four hips (8%) had missing height value and were replaced using multiple imputation. Importantly, 13 hips in 8 patients were excluded because they were IHDI grade 2, and none required femoral shortening. A comparison of the femoral shortening group and the control group is presented in Table 1. Side was the only factor that did not show statistical significance in the unadjusted association. Most of the femoral shortening group, 116 hips (97%), Which had a higher median age, underwent pelvic osteotomy after performing femoral shortening compared to 400 hips (93%) in the second group (*p* = 0.119).

**Table 1:** Participant characteristics at baseline

Characteristic	All Hips (*n* = 548)	femoral shortening (*n* = 119)	No femoral shortening (*n* = 429)	Odds ratio	95% CI	*P* Value
Median age (IQR), months	23 (19–32)	39 (31–52.5)	21 (19–26)	1.11	1.09–1.14	< 0.001
Female sex, %	461 (84)	91 (76)	370 (86)	1.93	1.16–3.20	0.015
Left side, %	295 (54)	67 (56)	228 (53)	1.14	0.75–1.71	0.612
Mean BMI (range)	15.61 (8.41–25.97)	14.89 (10.65–25.97)	15.81 (8.41–24.85)	0.87	0.80–0.94	< 0.001
IHDI 4, %	461 (84)	118 (99)	343 (80)	29.59	4.08–214.80	< 0.001

*IQR* interquartile range, *BMI* body mass index, *IHDI* international hip dysplasia institute

After logistic regression, starting with the full model and eliminating backwards, age, male gender, and IHDI grade were retained in the final model (Table 2). The regression coefficients were multiplied by the shrinkage factor of 0.97. The probability of femoral shortening can be calculated by routinely available predictors using the following formula: 1/(1 + exponential (−(− 7.11 + age in months * 0.10 + Male gender * 0.62 + IHDI grade 4 * 2.64))), where 1 in the equation is used for males and IHDI grade 4, 0 is inserted for Females and IHDI grade 3. For example, a 17-month old girl with IHDI grade 3 dislocation would have only a 0.4% probability of needing femoral shortening. In contrast, a 77-month old boy and IHDI grade 4 has a probability of femoral shortening as high as 98%.

**Table 2:** The model for the probability of femoral shortening in pediatric DDH surgery

Predictors	Odds ratio	95% CI	P Value	VIF
Age in months*	1.11	1.08–1.13	0.001	1.003
Male gender	1.89	0.10–3.60	0.052	1.002
IHDI grade IV vs III	15.44	2.05–116.21	0.008	1.000

*DDH* developmental dysplasia of the hip, *IHDI* international hip dysplasia institute, *VIF* variance inflation factor, (*) per unit change

**Fig. 1:**
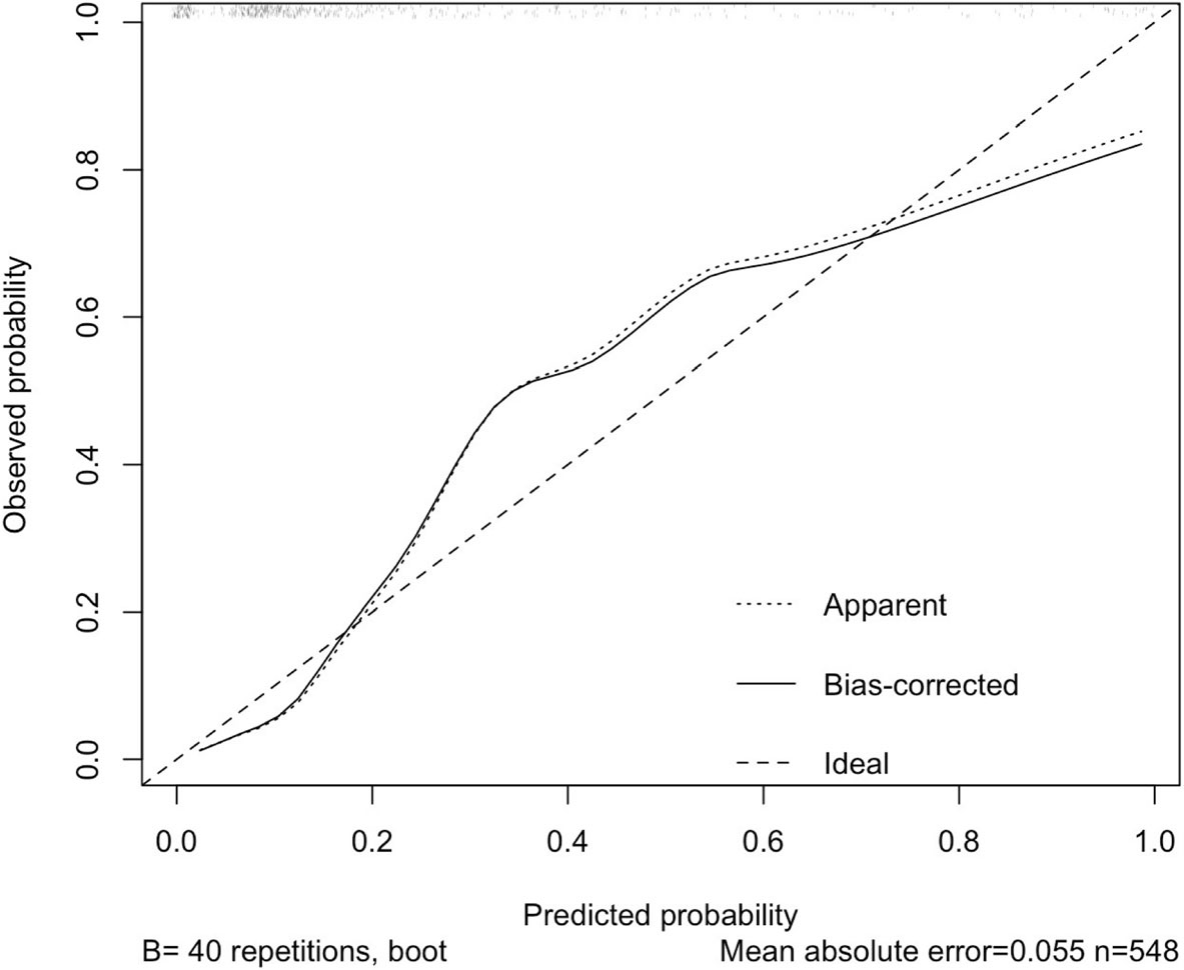
The calibration plot

**Fig. 2:**
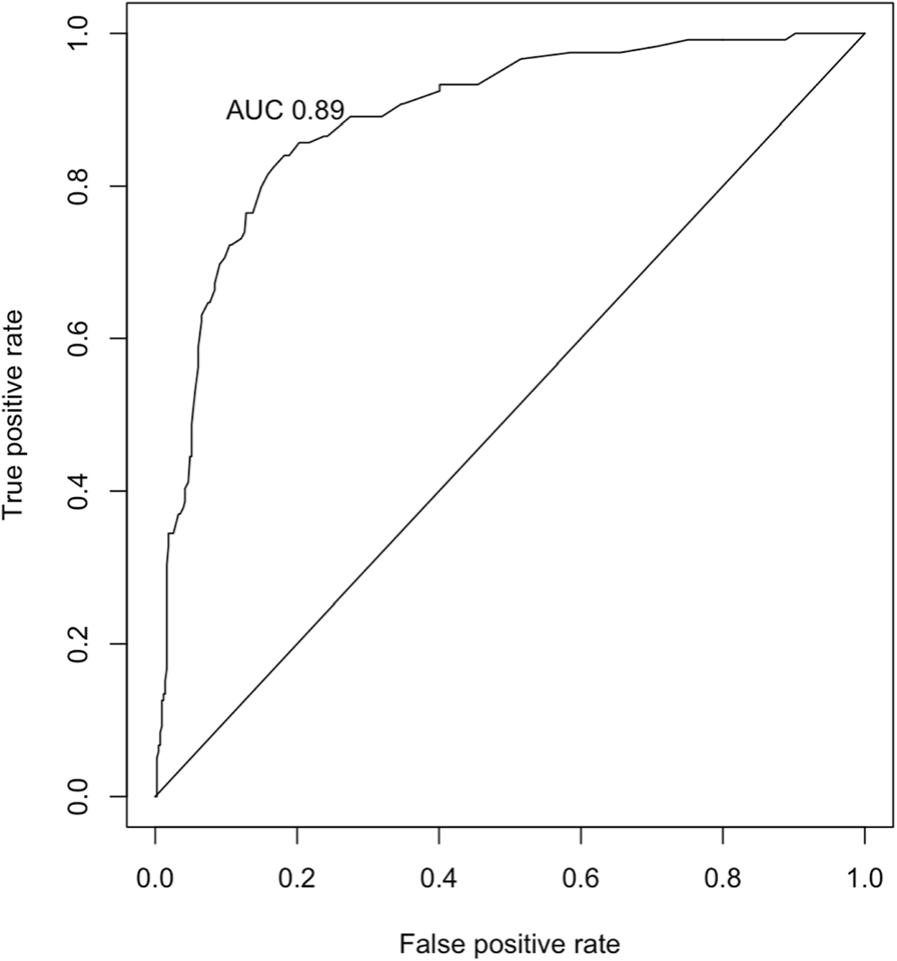
The receiver operator characteristic (ROC) curve, the area under the curve (AUC) 95% confidence interval is (0.86 to 0.92)

The VIF was low for all predictors, which indicates minimal multicollinearity (Table 2). The performance measure, calibration that illustrates the distribution of the predicted probabilities of hips with and without femoral shortening is presented in Fig. 1. The line is close to the 45 degrees perfect straight line with a slight underestimation in the middle range and mild overestimation in the higher range. Discrimination, which is the model’s ability to differentiate between hips that do or do not experience femoral shortening, is shown as an ROC curve in Fig. 2. AUC was 0.89, which reflects a strong discriminative ability of the model.

The documented complications are listed in Table 3. Except for deep infection, there was no difference in the crude comparison. However, multivariable analysis revealed that recurrence of instability is associated with older age (coefficient = 0.003, standard error = 0.0009, *p* <  0.001) and the lack of femoral shortening (coefficient = − 0.07, standard error = 0.028, *p* = 0.015). Avascular necrosis was not influenced by femoral shortening.

**Table 3:** Complications

	All Hips (*n* = 548)	Femoral shortening (*n* = 119)	No Femoral shortening (*n* = 429)	*P* Value	Additional surgeries
Median Follow-up (IQR), months	27 (13–48.25)	25 (12–40.5)	28 (13–54)	0.096	
Recurrent displacement, %	31 (5.7)	6 (5)	25 (5.8)	0.917	21 hips underwent revision
Avascular necrosis, %	30 (5.5)	5 (4.2)	25 (5.8)	0.644	One patient had greater trochanter transfer
Cast related fractures, %	12 (2.2)	1 (0.8)	11 (2.6)	0.477	One patient had operative fixation of tibia fracture and one femur fracture reduced under general anaesthesia
Full thickness pressure ulcers, %	2 (0.4)	0	2 (0.5)	1	
Unplanned dirty cast change, %	7 (1.3)	1 (0.8)	6 (1.4)	1	
Deep surgical site infection, %	4 (0.7)	3 (2.5)	1 (0.2)	0.034	3 were debrided once, one hip required several surgeries
Superficial surgical site infection, %	1 (0.2)	0	1 (0.2)	1	
Other early postoperative infections	16 (2.9)	4 (3.4)	12 (2.8)	0.759	
persistent stifness	6 (1.1)	1 (0.8)	5 (1.2)	1	Three hips had manipulation under anaesthesia without substantial improvement
Leg length discrepancy (> 2 cm)	3 (0.5)	1 (0.8)	2 (0.5)	0.521	
Neurological injury	2 (0.4)	0	2 (0.5)	1	One femoral nerve transection and common peroneal nerve entrapment recovered after K-wire removal and cast change

*IQR* interquartile rang

## Discussion

This study established and internally validated the first prediction model for femoral shortening in pediatric DDH surgery. Age, male sex, and IHDI grade 4 were the independent predictors. Both AUC and visual representation of the calibration presented here indicates a useful model [15]. These findings are in agreement with those of Sankar et al., who found in a study of 72 hips that femoral head superior displacement and age older than 3 years are associated with femoral shortening [12].

Age was significantly higher in femoral shortening patients, even after adjustment. Klisic et al. performed femoral shortening routinely after the age of 5 [4]. Dimitrio et al. and Ryan et al. did femoral shortening for all patients older than 3 years [2, 16]. Femoral shortening was performed routinely by Erturk et al. on 49 hips of children between the ages of 2 and 5, and only one hip redislocated [17]. Ning et al. performed femoral shortening in all of the 864 developmentally dysplastic hips in children older than three, 27.4% of these patients developed AVN of grade II or higher based on the Kalamchi classification, and their redislocation rate was 1.6% [18]. The side of the operated hip did not affect the rate of femoral shortening.

Male gender was associated with a higher probability of femoral shortening, even when the influence of other variables was statistically controlled. This difference can be explained, at least in part by increased muscle and tendon elasticity in females [19, 20]. BMI was eliminated from the reduced model in the multivariable analysis, as it was significantly lower in the group that had a higher age. BMI tends to decrease after the age of 1 until the age of 5.5 [21]. Because pelvic osteotomy is done after the completion of femoral shortening, it also was not added to the full model as a potential predictor.

Hefti suggested that femoral shortening should “always” be added to open reduction if the upper ridge of the femoral metaphysis is higher than the triradiate cartilage, which is equivalent to IHDI 4 [22]. In a study done by Cordier et al. 10 out of 118 hips required femoral shortening, and in all of these hips, the ossification centre was above the superolateral margin of the acetabulum (Tonnis grade 4) [23]. In the current study, 1 out of the 87 (1%) IHDI 3 hips required femoral shortening, and a quarter (118/461) of the IHDI grade 4 hips required femoral shortening to achieve a stable intraoperative reduction. The IHDI classification reliability has been tested, and it was used in this study because it does not depend on the presence of an ossific nucleus, which may not be always present [24].

Interestingly, femoral shortening might be protective against the need for additional procedures when combined with OR here, which is consistent with what was reported by Gholve et al., Based on data from 49 hips and a minimum follow-up of five years [25]. The risk of recurrence is increased with age as described in a recent report [18]. Femoral shortening did not affect the risk of avascular necrosis in the current study. In an analysis of 26 hips that underwent open reduction and acetabuloplasty, Akgul et al. performed femoral shortening in 13 hips. Avascular necrosis occurred in 6 hips and was distributed evenly between the two groups [26]. The probability of deep surgical site infection was higher when femoral shortening is performed (Table 3), which is a biologically plausible finding.

The lack of external validation was a limitation in this study. The indication for femoral shortening is clear when the hip fails to reduce after open reduction but deciding to perform femoral shortening based on tight reduction alone is not standardized, and tension on the femoral head is influenced by the extremity position. Although femoral shortening was decided based on intraoperative assessment in here, surgeons may add femoral shortening based on preconceived ideas.

## Conclusion

In addition to age and superior displacement of the femoral head, male gender is an independent risk factor for needing femoral shortening. The developed model may thus prove helpful in the preoperative phase. Personalizing the probability of femoral shortening may also further optimize family education, operating room preparation and operative time utilization. Femoral shortening may reduce recurrence in selected patients at the expense of a higher chance of deep surgical site infection.

## Abbreviations

AIC: Akaike information criterion
AUC: Area under the curve
BMI: Body mass index
DDH: Developmental dysplasia of the hip
IHDI: International hip dysplasia institute
IQR: Interquartile range
ROC: Receiver operator characteristic
VIF: Variance inflation factor
